# Dream characteristics in a Brazilian sample: an online survey focusing on lucid dreaming

**DOI:** 10.3389/fnhum.2013.00836

**Published:** 2013-12-10

**Authors:** Sérgio A. Mota-Rolim, Zé H. Targino, Bryan C. Souza, Wilfredo Blanco, John F. Araujo, Sidarta Ribeiro

**Affiliations:** ^1^Brain Institute, Federal University of Rio Grande do NorteNatal, RN, Brazil; ^2^Physiology Department, Federal University of Rio Grande do NorteNatal, RN, Brazil; ^3^Sleep Laboratory, Onofre Lopes University Hospital, Federal University of Rio Grande do NorteNatal, RN, Brazil; ^4^Computer Science Department, State University of Rio Grande do NorteNatal, RN, Brazil

**Keywords:** lucid dreaming, dreams, nightmares, REM sleep, dream features

## Abstract

During sleep, humans experience the offline images and sensations that we call dreams, which are typically emotional and lacking in rational judgment of their bizarreness. However, during lucid dreaming (LD), subjects know that they are dreaming, and may control oneiric content. Dreaming and LD features have been studied in North Americans, Europeans and Asians, but not among Brazilians, the largest population in Latin America. Here we investigated dreams and LD characteristics in a Brazilian sample (*n* = 3,427; median age = 25 years) through an online survey. The subjects reported recalling dreams at least once a week (76%), and that dreams typically depicted actions (93%), known people (92%), sounds/voices (78%), and colored images (76%). The oneiric content was associated with plans for the upcoming days (37%), memories of the previous day (13%), or unrelated to the dreamer (30%). Nightmares usually depicted anxiety/fear (65%), being stalked (48%), or other unpleasant sensations (47%). These data corroborate Freudian notion of day residue in dreams, and suggest that dreams and nightmares are simulations of life situations that are related to our psychobiological integrity. Regarding LD, we observed that 77% of the subjects experienced LD at least once in life (44% up to 10 episodes ever), and for 48% LD subjectively lasted less than 1 min. LD frequency correlated weakly with dream recall frequency (*r* = 0.20, *p* < 0.01), and LD control was rare (29%). LD occurrence was facilitated when subjects did not need to wake up early (38%), a situation that increases rapid eye movement sleep (REMS) duration, or when subjects were under stress (30%), which increases REMS transitions into waking. These results indicate that LD is relatively ubiquitous but rare, unstable, difficult to control, and facilitated by increases in REMS duration and transitions to wake state. Together with LD incidence in USA, Europe and Asia, our data from Latin America strengthen the notion that LD is a general phenomenon of the human species.

## Introduction

Dreams are characterized by sensory, perceptual and cognitive experiences during sleep, usually presenting a strong emotional imprint, and being interpreted as if they were real, i.e., without concern about their bizarreness (Hobson et al., 2000). However, during lucid dreaming (LD), subjects know they are dreaming during the dream, and may control oneiric content (Laberge et al., 1981a; Laberge, 1988), an exception to the rule that dreaming is necessarily an experience concurring with no rational judgment. In Western history, Aristotle’s book *On sleep and sleeplessness* is one of the first known references on the possibility of becoming aware of the dream while dreaming. In *The interpretation of dreams* Freud (1900) stated: “… there are people who, during the night, know they are sleeping and dreaming, and then are able to consciously change their dreams”. Van Eeden (1913), who coined the term “lucid dream”, explains that during this kind of dream “…the reintegration of the psychic functions is so complete that the sleeper remembers day-life and his own condition, reaches a state of perfect awareness, and is able to direct his attention, and to attempt different acts of free volition”. More recently, Voss et al. (2013) compared lucid and non-lucid dreams and created a scale based on factors involved in becoming lucid during dreaming: insight, control over thoughts and actions, logical thoughts, access to the mnemonic elements of waking life, and positive emotions.

Neurophysiological studies on LD began with Hearne (1978) and were advanced by Laberge (1980), who developed a technique that consists of instructing subjects to convey an objective signal through ocular movements (e.g., two consecutive left-right turns) (Laberge et al., 1981a) or respiration control (e.g., to breathe rapidly) (Laberge and Dement, 1982) whenever they became lucid while dreaming. This is possible because ocular and respiratory muscles are not in atonia during rapid eye movement sleep (REMS; Aserinsky and Kleitman, 1953; Dement and Kleitman, 1957), the sleep stage most associated with dreaming (Hobson et al., 2000).

Intriguingly, LD prevalence varies substantially among countries: 26% of a representative sample from Austria (*n* = 1,000) reported having a LD at least once in life (Stepansky et al., 1998), while in Germany (*n* = 919), 51% said so (Schredl and Erlacher, 2011). College students in Japan, United States, Holland, Germany and China reported LD prevalences of 47% (*n* = 153) (Erlacher et al., 2008), 71% (*n* = 268) (Palmer, 1979), 73% (*n* = 189) (Blackmore, 1982), 82% (*n* = 439) (Schredl and Erlacher, 2004), and 92% (*n* = 348) (Yu, 2008), respectively. Possible reasons for this discrepancy across studies may rest on the usage of different LD definitions, uncontrolled variability in the volunteers’ understanding of these definitions (Erlacher et al., 2008), age differences of the samples (Voss et al., 2012), or variability in other sociocultural aspects, such as the practice of meditation, which is associated with an increased frequency of LD reports (Gackenbach, 1981, 1990; Hunt, 1991).

To our knowledge, there are to date no studies about dream features among Brazilians, nor studies regarding LD prevalence among Latin Americans. Moreover, there is a lack of knowledge regarding LD characteristics in this population, such as number of episodes experienced in lifetime, ability to control oneiric content, episode duration, and facilitating factors of occurrence. It is therefore important to obtain data on these LD features to compare with other populations, or with laboratory studies, such as Laberge et al. (1986), who observed that LD lasted about 2 min in average, but could reach up to 50 min. Thus, to fill this gap, we set out to investigate the characteristics of regular dreaming and LD through an online questionnaire in a sample of 3,427 Brazilian subjects. To facilitate our respondents understanding the difference between lucid and non-lucid dreams, in the present study we used the following sentence: “As bizarre as dreams are, we tend to believe that what is happening during the dream is real. However, during a special kind of dream called lucid dreaming, we are sure to be dreaming during the dream, and we may come to control dream content”. The investigation of LD was accompanied by an assessment of general dream features that may influence LD. For instance, remembering more dreams in general is likely to increase the chances of experiencing LD (Laberge and Rheingold, 1990), and therefore we investigated the frequency of dream recall. We further interrogated about bedroom elements that may be incubated in dreams, because incubation of auditory (Laberge et al., 1981b) or visual (Laberge et al., 1988) stimuli into REMS may act as a cue for the subject to become lucid during dreaming. Finally, we investigated recurrent dreams and nightmares, since both may work as a “dream sign”, which facilitates dream lucidity (Saint-Denys, 1982; Tholey, 1988; Laberge and Rheingold, 1990; Schredl and Erlacher, 2004).

## Materials and Methods

### Subjects

**Figure 1:**
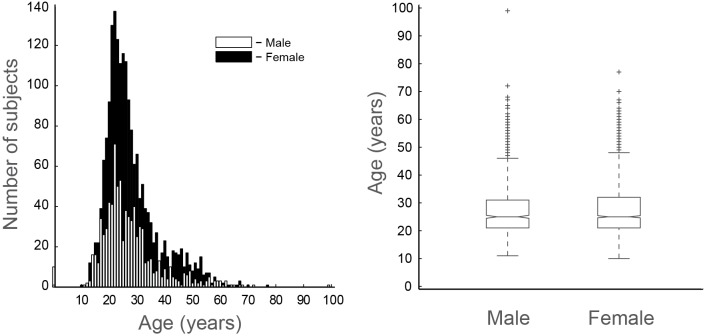
**Epidemiological characteristics of the population sample.** Age distribution (left; white bars = male, black bars = female) and boxplot of ages within genders (right). Outliers indicated by crosses.

The study was approved by the Research Ethics Committee of the Federal University of Rio Grande do Norte (permit #061/2008). As stated by the Ethics Committee, all subjects (*n* = 3,909) completed an online informed term of consent before completing the questionnaire. Subjects were invited to respond the questionnaire directly by email, or indirectly by online social network services or TV program ads. Subjects who did not answer a given question were excluded from the analysis of this question. We also excluded the subjects who answered less than 90% of the first part of the questionnaire (final sample = 3,427 subjects; median age = 25 years, 56% female and 24% male, 20% did not inform gender) (Figure 1). In order to check whether there is an age difference between men and women, we normalized the distributions by the maximum value, and also by *Z*-Score, since many more women answered the questionnaire. Then, the distributions were compared using the Kolmogorov-Smirnov test (controlled by a bootstrap surrogate technique). We also investigated a possible age group effect on the questionnaire responses.

### Questionnaire

The questionnaire was divided in two parts: the first part consisted of 10 questions about regular dreams, while the second part involved 10 questions about LD. To facilitate and standardize the subjects’ understanding of the difference between lucid and non-lucid dreams, we provided the following explanatory sentence at the onset of the survey: “As bizarre as dreams are, we tend to believe that what is happening during the dream is real. However, during a special kind of dream called lucid dreaming, we are sure to be dreaming during the dream, and we may come to control dream content”.

The first part was divided in 4 radio questions (that admit only one answer), 1 check-box question (that admits none, one or more answers) and 5 table questions. These table questions were divided by dream items according to frequency of occurrence: never, very rare (once a year), rare (once a month), frequent (once a week), very frequent (almost every day), and always (every day); for the sake of synthesis, we present the results of the last three answers grouped. The second part of the questionnaire was divided in 7 radio questions, 2 check-box questions and 1 mixed (radio and check-box) question. Details about the original questionnaire can be found at: http://www.cb.ufrn.br/sonho/sonholucidoform.html. A version translated to English is included in the Supplementary Material.

### Data acquisition and pre-processing

Questionnaires were created using HTML and PHP language and were available to be answered in a website of the Federal University of Rio Grande do Norte.^1^ After the questionnaire is filled, the answers were automatically sent to an email account and then converted to MATLAB format. We dropped out 8 questions that were not directly important to our objective, and of the 12 questions that remained, 4 are ordinals—to facilitate correlation analysis interpretation, we transformed all these questions in a direct crescent order.

### Descriptive and correlation analysis

**Figure 2:**
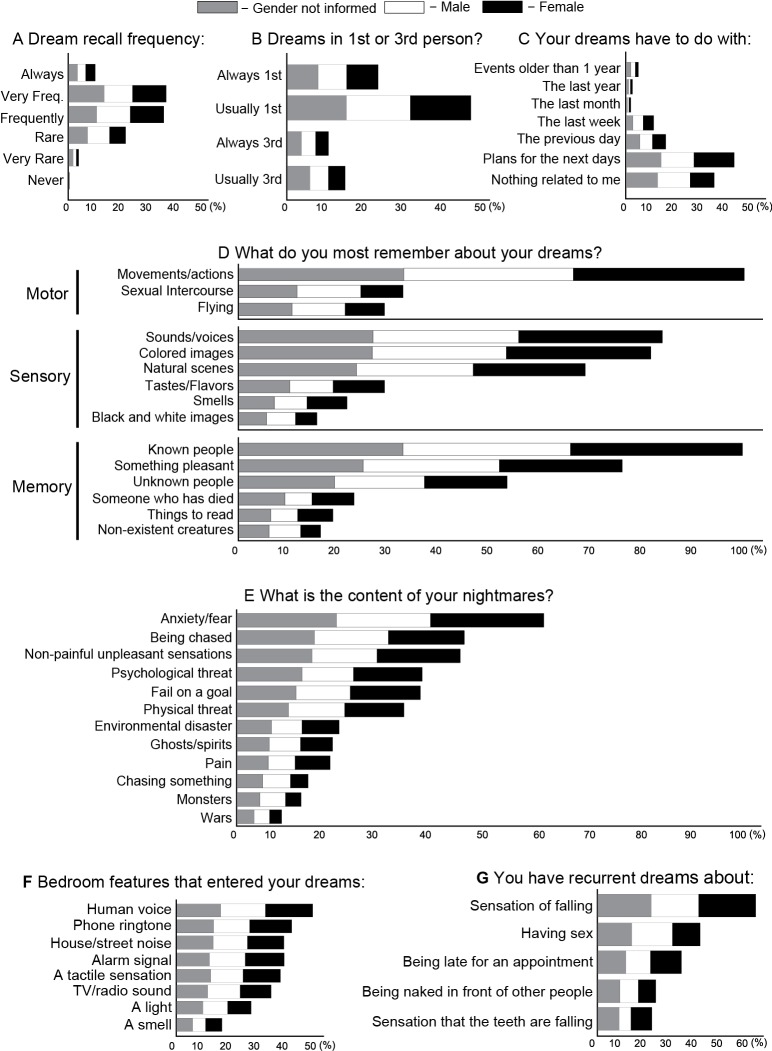
**Frequency and content of dreams and nightmares for female (black), male (white), gender not answered (gray). (A)** Frequency of dream recall. **(B)** Frequency of dream content according to first or third person point of view. **(C)** Dream content according to time: plans, recent or old memories. **(D)** Dream features. **(E)** Nightmares features. **(F)** Sleep environment elements that incubate into dreams. **(G)** Recurrent dream features.

**Figure 3:**
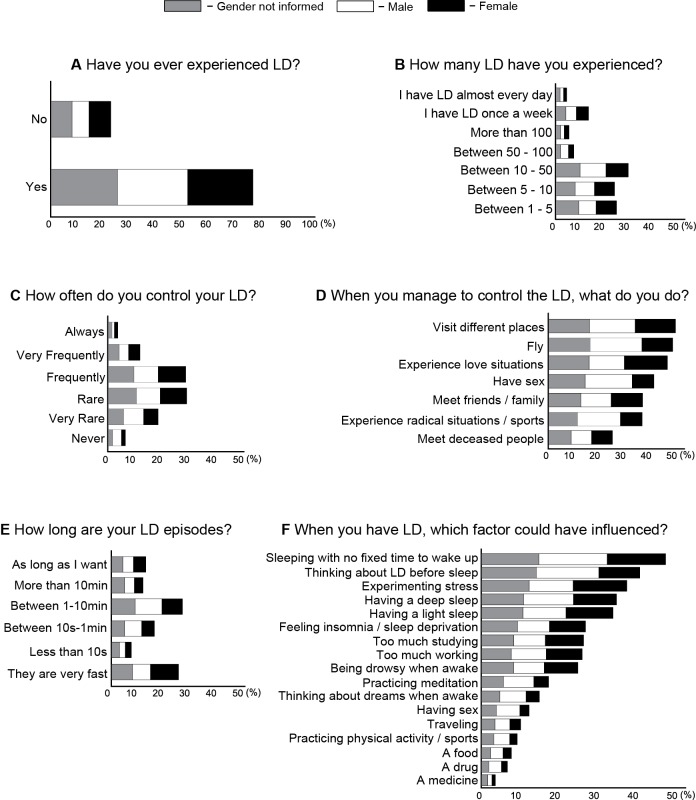
**Frequency and content of LD for female (black), male (white), gender not answered (gray). (A)** Percentage of LD report for at least once in lifetime. **(B)** Number of LD episodes recall. **(C)** Frequency of LD control. **(D)** Things to do during LD. **(E)** LD episodes duration. **(F)** Factors that may have facilitated LD occurrence.

**Figure 4:**
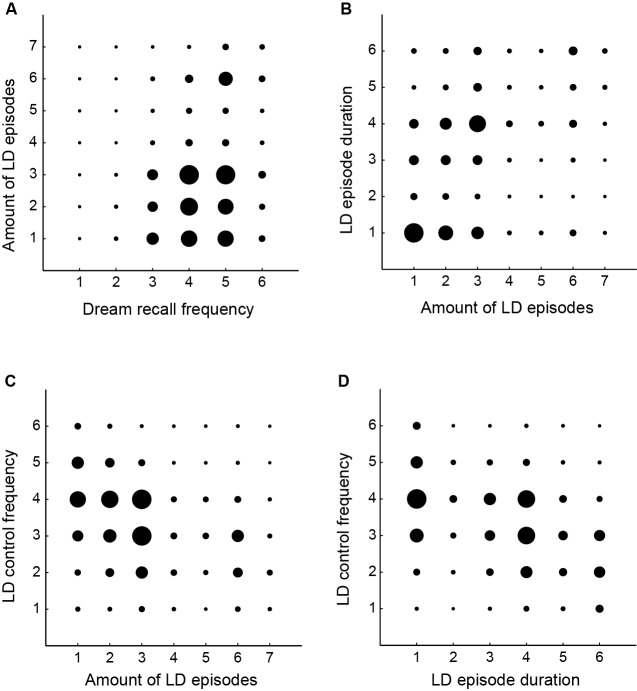
**Correlations between dreaming and LD features. (A)** Amount of LD episodes and dream recall frequency (*r* = 0.20, *p* < 0.01). **(B)** Amount of LD episodes and LD episode duration (*r* = 0.34, *p* < 0.01). **(C)** Amount of LD episodes and LD control frequency (*r* = −0.33, *p* < 0.01). **(D)** LD control frequency and LD episode duration (*r* = −0.38, *p* < 0.01). The size of the black circles is associated with the number of subjects that responded to the pair of answers for both questions. The amount of LD episodes were measured on a 7-point rating scale: 1 = between 1–5, 2 = between 5–10, 3 = between 10–50, 4 = between 50–100, 5 = more than 100, 6 = every week, 7 = almost every day. Dream recall frequency was measured on a 6-point rating scale: 1 = never, 2 = very rare (once a year), 3 = rare (once a month), 4 = frequently (once a week), 5 = very frequently (almost every day), 6 = always (every day). LD episode duration was measured on a 6-point rating scale: 1 = very fast, 2 = < 10 s, 3 = 10 s–1 min, 4 = 1–10 min, 5 = > 10 min, 6 = the time I want. LD control frequency was measured on a 6-point rating scale: 1 = never, 2 = very rare, 3 = rare, 4 = frequently, 5 = very frequently, 6 = always.

For dreams (Figure 2) and LD (Figure 3), we plotted the percentage only for those who answered that specific question (male in white, female in black and “gender not informed” in gray bars sum 100%). For ordinal questions (Figure 4), we performed a Spearman correlation analysis. Dream recall frequency was measured on a 6-point rating scale: 1 = never, 2 = very rare (once a year), 3 = rare (once a month), 4 = frequently (once a week), 5 = very frequently (almost every day), 6 = always (every day). LD frequency was measured on a 7-point rating scale: 1 = between 1–5, 2 = between 5–10, 3 = between 10–50, 4 = between 50–100, 5 = more than 100, 6 = every week, 7 = almost every day. LD duration was measured on a 6-point rating scale: 1 = very fast, 2 = less than 10 s, 3 = between 10 s–1 min, 4 = between 1–10 min, 5 = more than 10 min, 6 = the time the subject wants. LD control frequency was measured on a 6-point rating scale: 1 = never, 2 = very rare (once a year), 3 = rare (once a month), 4 = frequently (once a week), 5 = very frequently (almost every day), 6 = always (every day).

## Results

### Subjects

3,909 voluntaries responded to the survey, but we excluded those who answered less than 90% of the dream questionnaire (see Section Materials and Methods). In our final sample (*n* = 3,427), 56% were female, 24% were male and 20% did not answer the gender. The median age was 25 years (Figure 1). Since many more women attended the survey, and in order to investigate whether there is an age difference among gender, we normalized the distributions, and performed a Kolmogorov-Smirnov test, which showed that both distributions are statistically indistinguishable (KS: *H* = 0, *p* = 0.2056). We also investigated a possible age group effect on the responses, but no significant differences were observed.

### Dream and nightmare features

Subjects who did not answer a given question were excluded from the analysis of this question (see Section Materials and Methods). We found that 34.1% of the subjects remembered dreams frequently (1 or 2 times per week), 33.2% almost every day, 19.8% about twice a month, 9.2% every day, 3.4% once a year and 0.1% less than once a year (Figure 2A). With regards to the dreaming point of view, 23.8% of the respondents observe the dream always in first person, 46.2% usually in first person, 11.7% always in third person and 15.3% usually in third person (Figure 2B). A total of 37.8% of the subjects reported that their dream was mostly associated with plans for upcoming days and 30.7% claimed that their dreams have nothing to do with them. For 14.2% of the respondents, dreams were associated with the previous day, for 8.8% with the last week, for 4.7% with events that happened for more than one year, for 2.1% with the last year and for 1.8% with the last month (Figure 2C).

We also observed that dream content mainly involved movements/actions (93.3%), known people (92.9%), sounds/voices (78.5%), colored images (76.3%), something pleasurable (70.7%) and natural scenes (63.9%). The less common features were unknown people (49.7%), sexual intercourse (30.4%), flying (26.9%), tastes/flavors (26.8%), someone who has died (21.2%), a smell (20.0%), things to read (17.4%), nonexistent creatures (15.0%), black and white images (14.3%) (Figure 2D). During nightmares, it is more frequent to experience the presence of anxiety/fear (65.5%), being chased (48.5%), non-painful unpleasant situations (47.6%), psychological threat (39.5%) frustration or failure in a goal (39.1%) and physical threat (35.6%). The less common nightmare features were environmental disasters (21.8%), ghosts/spirits (20.4%), pain sensation, (19.8%), chasing something (15.1%), monsters (13.6%) and wars (9.4%) (Figure 2E).

The main sleep room or environmental stimuli that incubate into dreams were the voice of someone (47.6%), phone ring (40.1%), alarm clock (37.5%), house/street noise (37.4%), a tactile sensation (36.2%), TV/radio sounds (32.9%), a light (25.9%), a smell (15.7%) (Figure 2F). The recurrent dreams content were mainly associated with a dream with a sensation of being falling (55.2%), having sex (35.6%), being late for an appointment (29.2%), being naked in front of people (20.2%) and feel the teeth falling out (18.8%) (Figure 2G).

### Lucid dreaming features

We observed in our sample that 77.2% of the subjects had already experienced at least one LD episode in their whole lifetime (Figure 3A). With respect to the number of LD episodes, 27.2% had experienced between 10–50 episodes, 22.8% between 1–5, 22.1% between 5–10, 12.2% have LD every week, 6.6% had between 50–100, 4.8% had more than 100 episodes, and 3.9% have LD almost every day (Figure 3B). With regards to the frequency of controlling LD content, 29.7% of the respondents control LD rarely, 29.3% frequently, 18.8% very frequently, 12.0% very rare, 6.4% always and 3.6% never (Figure 3C). Whenever subjects are able to control LD, 47.6% choose to visit different places, 46.7% to fly, 44.6% to experience love situations, 39.5% to have sexual intercourse, 35.3% prefer to meet friends, 35.2% to experience radical situations, and 23.9% to meet deceased people (Figure 3D).

With respect to LD episode duration, 26.7% report that LD takes between 1–10 min, but 25.2% tend to wake up after realizing the LD. For 16% LD takes between 10 s and 1 min, and for 12.8% LD takes the time the dreamer wants. For 11.7% LD takes more than 10 min and for 7.3% less than 10 s (Figure 3E). The facilitating factors for LD occurrence were related to: sleep without a fixed time to wake up (38.3%), think about LD before sleep (32.8%), experiencing stress (30.1%), have a deep (28.1%) or a light sleep (27.3%), insomnia (21.5%), too much study (21.1%), too much work (20.9%), be sleepy when awake (20.0%), practice meditation (13.9%), think about dreams during waking (11.9%), have sex (9.3%), travel (8.1%), practice physical activity (7.3%), a food (6.1%), a drug (5.3%), a remedy (2.8%) (Figure 3F). LD frequency was positively correlated with dream recall frequency (*r* = 0.20, *p* < 0.01— Figure 4A), with LD episode duration (*r* = 0.34, *p* < 0.01— Figure 4B) and negatively with LD control frequency (*r* = −0.33, *p* < 0.01— Figure 4C). LD control frequency was negatively correlated with LD episode duration (*r* = −0.38, *p* < 0.01— Figure 4D). LD report (at least once in lifetime) was most common in male (75%) than in female (68%) (*χ*^2^ = 10.2, *p* = 0.001).

## Discussion

One important limitation of our study, intrinsic to an online survey, is the lack of information about the physiological state underlying each dream report. Dreams are not restricted to REMS (Hobson et al., 2000; Solms, 2000), and therefore the data collected likely reflect a mix of consciousness states. Irrespective of this caveat, we observed that dream reports were mainly related to plans for the next days, but were also associated with memories of the previous days, months or years (Figure 2C). Nightmare reports dealt mainly with situations somewhat likely to occur in everyday life, such as experiencing anxiety and fear, being physically/psychologically threatened, and feeling unpleasant sensations or frustrations; in contrast, unlikely events such as suffering environmental disasters, meeting non-existent creatures such as monsters, ghosts or spirits, chasing someone/something, or being in a war were less reported as nightmare contents (Figure 2E). While these results seem to support the notion of day residue (Freud, 1900), the hypothesis is limited by the fact that pain, a relatively common wake experience, is not frequent in dream records, as found by Zadra et al. (1998b) and also here (Figure 2E). On the other hand, the results are more compatible with the theory that nightmares (Revonsuo, 2000), and perhaps all dreams (Ribeiro and Nicolelis, 2006; Mota-Rolim and Araujo, 2013), constitute adaptive behavioral simulations related to the social, psychological and biological fitness of the dreamer. Specifically regarding LD, we observed that it is relatively ubiquitous although infrequent, unstable, and difficult to control (Figure 3). Adding Latin American data to prior assessments of LD prevalence among North Americans (Palmer, 1979), Europeans (Blackmore, 1982; Stepansky et al., 1998; Schredl and Erlacher, 2004, 2011) and Asians (Erlacher et al., 2008; Yu, 2008), our results strengthen the notion that LD is a general phenomenon of the human species.

We initially investigated non-lucid dreams, and observed that most respondents claimed to remember dreams once or twice a week (Figure 2A), in accordance with similar studies on dream recall frequency (Herman and Shows, 1983; Schredl et al., 2003; Nielsen et al., 2006). The dream content, according to subjective point of view, was classified as first person dreams (active dreams “from within”, in which the subject makes decisions and acts at will), or as third person dreams (passive dreams, in which the dreamer participates “from without” as an observer, spectator or just another dream character). We found that subjects tended to dream more in first person than in third person (Figure 2B), indicating that self-consciousness is preserved in most dreams. We also observed that dreams were related to memories of previous days, weeks, months and even years (Figure 2C), which is in accordance with Freudian theory of “day residue” (Freud, 1900). Surprisingly, dreams associated more with plans for the next day, suggesting that the oneiric content relates with simulations of future scenarios (Revonsuo, 2000). However, about one third of subjects reported that their dreams had nothing to do with their lives (Figure 2C), supporting the existence of stochastic influences over dreaming (Hobson and McCarley, 1977; Foulkes, 1985; Hobson et al., 2000), which restructure memory traces so strongly that mnemonic activation ends up not being recognizable by the dreamer (Ribeiro and Nicolelis, 2006).

The general dream content (Figure 2D) mainly involved movements and actions, known people, colored images and sounds/voices, in accordance with previous studies (McCarley and Hoffman, 1981; Zadra et al., 1998a) and likely reflecting the sensorimotor repertoire of our daily life. Smells are unlikely to be present in dreams (Figure 2D), which is in accordance with Hobson et al. (2000). Reading was also rare during dreams (Figure 2D), which could be due to a low blood flow in the frontal cortex during REMS (Maquet et al., 1996) that may impair attention (Tsakiris et al., 2007) and working memory related tasks (Baddeley, 1992; Hobson and Stickgold, 1994; Revonsuo and Salmivalli, 1995; Baddeley and Della Sala, 1996; Hobson, 1997; Courtney et al., 1998).

During nightmares (Figure 2E), subjects reported mainly anxiety and fear, which is in accordance with a previous study (Merritt et al., 1994). Other frequent nightmare contents were being stalked, frustration or failure to reach a goal, and psychological or physical threat, in this order of prevalence. The less common nightmares were related to environmental disasters, ghosts, feeling pain, chasing something/someone, monsters and war, respectively. The threat-simulation theory proposed by Revonsuo (2000) postulates that dreams and nightmares are meant to simulate situations that can happen in the real world. This is corroborated by the observation that all sensory modalities are present in dreams with a frequency comparable to that of wakefulness, according to Zadra et al. (1998a) and also observed here (Figure 2D). Emotions during dreaming are mainly fear or anxiety (Snyder, 1970), as found here (Figure 2E). Aggression is the most frequent form of social interaction during dreaming, and dreamers are primarily victims (Hall and Van De Castle, 1966). Consistent with this, we also observed that it is much more common to being stalked than to chase something or someone (Figure 2E). The limbic activation during REMS, especially in the amygdala (Maquet et al., 1996; Braun et al., 1998) would be the neural correlate of threat-simulation (Revonsuo, 2000).

To Revonsuo (2000), the threat-simulation theory is based on the fact that the prehistoric environment—in which the human brain evolved—included frequent dangerous events, such as animals’ and/or other human groups’ threats in competition for territory or food, which challenged the reproductive success of the hunter-gatherers, and therefore represented important selection pressures on those populations. This is observed by the increased presence of such content in young children dreams (whose brain has not had a chance to adjust to contemporary society) and its gradual decline into adulthood (Strauch, 1996). Gregor (1981) analyzed the content of 385 dream reports obtained among the Mehinaku Indians (from Brazil), and observed that their dreams contained significantly more physical aggression (mostly from animals) in comparison with a sample of townspeople. A similar result was observed by Calvin Hall in the early 1930’s, among the Yir Yoront, a native population of Australia (*apud* Domhoff, 1996).

We further investigated the environmental stimuli in the sleeping room able to incubate into dreams. The most reported sensory modality to enter dreams was the auditory one, such as the voice of someone, phone ring, alarm clock, and house or street noise; the less frequent were tactile stimuli, light and smells (Figure 2F), which is in accordance with previous studies (Freud, 1900; Laberge et al., 1981b, 1988; Carskadon and Herz, 2004). With regard to recurrent dreams, we observed that the most reported content was dreaming with the sensation of falling (Figure 2G), which may be attributed to a rapid decline in muscle tone during sleep (or REMS) onset. Having sex, being late for an appointment, or being naked in front of other people are frequent contents (Figure 2G), perhaps because desires and fears play a major role in shaping dreams (Freud, 1900; Revonsuo, 2000). Another frequent content of recurrent dreams is teeth loss, in line with previous reports (Schredl et al., 2004; Zadra et al., 2006). The explanation for this kind of recurrent dream remains speculative: Lorand (1948) believes that it is associated with masturbation in men, parturition in women or regression to childhood, while Schneck (1956, 1967) postulates a link with the fear of growing old (*apud* Schredl et al., 2004).

Regarding LD, we observed that 77,2% of our sample already had experienced LD at least once in lifetime (Figure 3A). However, LD prevalence varies substantially among different populations, ranging from 26% (Stepansky et al., 1998) to 92% (Yu, 2008). We believe that two factors may contribute to the discrepancy in LD prevalence across studies: (1) researchers provided different definitions of LD to the respondents, and (2) the LD concept itself is difficult to understand, especially for those who are not used to remember or talk about dreams. In our study, the questionnaire was applied through the internet; to minimize this limitation, we tried to provide a clear definition of LD (see Section Materials and Methods). Moreover, LD questions came only after the questions about non-lucid dreaming; this may have helped subjects to better understand the differences between these kinds of dream (see Supplementary Material). It should be noted that this relatively new field still lacks a consensual standard on the definition of Lucid Dreaming. The study by Voss et al. (2013), which investigated consciousness features during dreaming, was published after our data was collected, and thus we could not use their comprehensive LD definition in our survey. We also believe that epidemiological characteristics of the analyzed populations may explain the different prevalence of LD in distinct samples, such as age (Voss et al., 2012) and meditation practice (Gackenbach, 1981, 1990; Hunt, 1991), for example.

We found a correlation between dream recall frequency and LD frequency (Figure 4A), which is in accordance with previous studies (Blackmore, 1982; Wolpin et al., 1992; Schredl and Erlacher, 2004, 2011; Voss et al., 2012). In accordance, Laberge and Rheingold (1990) argue that remembering more dreams in general should increase the chances of remembering LD. In the present study, we observed that LD was more frequent among males than females. Most studies reported no differences in LD frequency between genders (Gruber et al., 1995; Stepansky et al., 1998; Schredl and Erlacher, 2004), but one study reported that LD recall was higher in women (Schredl and Erlacher, 2011). In our survey, women were much more participative (Figure 1), and it is possible that the men who answered the questionnaire were on average more likely to have experienced LD than the general male population, which could have biased our results.

The report of having experienced at least one LD episode was frequent (Figure 3A), but at the same time LD was largely non-recurrent; most of the people had less than 10 episodes in their whole lifetime (Figure 3B). Based on the observations that LD occurs predominantly during REMS (Brylowski et al., 1989; Laberge and Rheingold, 1990) and most people present REMS every night, an intriguing issue is why LD is so uncommon. We have previously proposed that a likely explanation for this discrepancy is that there exists more than one kind of REMS, and that the specific kind of REMS during which LD occurs is rare, with EEG spectral features that differentiate it from non-lucid REMS (Mota-Rolim et al., 2010). Consistent with this, early studies reported that the level of lucidity relates to the overall power in the alpha band (8–12 Hz) (Ogilvie et al., 1982; Tyson et al., 1984). However, more recent work found increased EEG power within the beta band in the parietal area (Holzinger et al., 2006), and the gamma band (peaking around 40 Hz) in the frontal region during LD (Hobson, 2009; Voss et al., 2009). Using cognitive tasks and a dream diary, Neider et al. (2011) observed that subjects who performed better on a task that engages the ventromedial prefrontal cortex exhibited more lucidity reports. This was not true for a task related to the dorsolateral prefrontal cortex (Neider et al., 2011). Therefore, there is evidence to suggest that LD present different spectral characteristics than non-LD, despite the disagreement with regard to the brain regions and frequency bands most related to LD. We recently suggested that different subjective experiences during LD could have different underlying neural substrates (Mota-Rolim et al., 2010). In accordance, Dresler et al. (2011) observed that performing hand movements during LD specifically elicits neuronal activation in the sensorimotor cortex.

We also observed that it is difficult to achieve full volitional control of LD (Figure 3C), which is typically ephemeral—the majority of our sample reported that LD subjective duration was below 1 min (Figure 3E). A laboratory-based study with experienced lucid dreamers found that LD (verified by eye-movement lucidity signal) lasted an average of 115 s (range from 5 s to 490 s), up to 50 min in length (Laberge et al., 1986). Although the data on LD duration is problematic, given the known distortion of time perception during dreaming, Dement and Kleitman (1957) described a temporal correspondence between dream and waking events. In this study, participants were randomly awoken 5 or 15 min after the onset of REMS. After waking up, subjects were asked whether they had dreamed for 5 or 15 min: in a total of 111 awakenings, the correct time estimation was observed in 83% of the reports. Other studies found similar results, such as Glaubman and Lewin (1977), and Hobson et al. (2000). Recent studies have suggested that time perception in LD is similar to wakefulness, but motor activity is slower (Erlacher and Schredl, 2004). We also found a negative correlation between LD control and LD duration (Figure 4D), suggesting that when subjects try to control LD they tend to wake up.

The factors that facilitated LD occurrence (Figure 3F) were related to sleep and dream features (e.g., sleeping without a fixed time to wake up, thinking about having a LD before sleeping, thinking about dreams during the day), negative stimuli (stress, too much study, too much work, or insomnia), positive stimuli (meditation practice, sexual intercourse, traveling, physical activity), among others (drug use, food intake). Consistent with our data, Laberge and Rheingold (1990) also observed that thinking about having a LD before sleeping may induce LD, indicating that LD occurrence is susceptible to suggestion. Sleeping without a fixed time to wake up may facilitate LD because it is associated with REMS (Brylowski et al., 1989), the sleep stage more related to dreaming (Hobson et al., 2000), which is prevalent in the last hours of sleep (Aserinsky and Kleitman, 1953; Dement and Kleitman, 1957). Stressful factors such as insomnia, sleep deprivation, excessive study and/or work, were also facilitating factors (Figure 3F). This could be due to an increase of REMS transitions into the waking state associated with stress (Kim and Dimsdale, 2007), which would support the hypotheses that LD could happen in the transition phase from REMS to waking. The important incidence of such transitions is pointed by Mahowald et al. (2011): “…even in normal subjects, the electrographic and neuronal activity transitions among states are gradual and variable, with the simultaneous occurrence or rapid oscillation of multiple state-determining markers indicating ongoing variability and fluctuation of state determination underscoring the fact that sleep is not a global, whole brain phenomenon”.

We confirmed the observation that meditation practice increases LD frequency (Gackenbach, 1981, 1990; Hunt, 1991; Figure 3F). A previous study found that long-term meditation practitioners have increased rapid eye movement density during REMS (Manson et al., 1997), which could be related to a higher LD frequency in these subjects. However, for Ogilvie et al. (1982), LD and meditation would be related by the increased power in the alpha band (8–12 Hz) observed in both mental states. Other authors believe that this correlation is associated with a greater mental control, which would emerge in both meditation practitioners and frequent lucid dreamers (Blagrove and Tucker, 1994; Blagrove and Hartnell, 2000). Buddhist monks from Tibet also developed the so-called “dream yoga”: this meditation technique is based on cognitive-behavior methods to induce LD direct from wakefulness (Laberge, 2003). We have not found references in literature with respect to others factors that facilitate LD occurrence.

To conclude, we believe that dreams may have acquired an adaptive function, acting as a simulation of the past (associated with memory), or the future (associated with plans and expectations) (Ribeiro and Nicolelis, 2006). From this point of view, dreams are mainly related to two forces: wishes, as Freud (1900) postulated, but also fears (Revonsuo, 2000). These are the elementary tenets of evolution: based on past experiences we desire the pleasant, but are also afraid of taking risks (Mota-Rolim and Araujo, 2013). As a special type of dream, our results indicate that LD is relatively common but not recurrent, often elusive and difficult to control. About three quarters of the Brazilian subjects in our sample reported having experienced at least one LD in their lifetime. Despite the variable prevalence of LD among different populations in Europe, Asia, North and now South America, our data strengthens the idea that LD is a general phenomenon of the human species. Since LD has been neglect by most neuroscientists and psychoanalysts, our results may call their attention to this important phenomenon.

Having performed an internet survey about dreaming, we are aware of the intrinsic methodological limitations of data reliability. First, it has no supervision and is prone to respondents’ exaggerations and/or understatements. Second, responses were collected through an online survey, thus yielding a biased sample, at the very least restricting it to people with internet access. Thus the conclusions drawn from our survey should not be taken at face value as representative of the whole population. It is also important to point out that since our study was not a laboratory-based dream investigation, we dealt with dream reports and what is remembered of them—not dream content collected immediately after awakening—especially because we asked for reports on dream content covering a wide time range, without distinction between recent and remote dreams. Other limitations include no data on subject occupation and LD entry state (from waking or from dreaming) (Laberge, 1988). Finally, it is important to point out that REMS dreams and LD are likely to be confounded with other states of consciousness not addressed in this survey, such as: (1) the physiological transition from the waking state to dreaming, and from dreaming to the waking state (hypnagogic and hypnopompic hallucinations, respectively); (2) during altered mental states such as hypnosis, trance etc.; and (3) pathologically, as in REMS behavior disorder and sleep paralysis, among others (Mahowald et al., 2011).

## Conflict of interest statement

The authors declare that the research was conducted in the absence of any commercial or financial relationships that could be construed as a potential conflict of interest.

## Footnotes

^1^http://www.cb.ufrn.br/
