# Supplementary material for: Dream characteristics in a Brazilian sample: an online survey focusing on lucid dreaming
Source: Front Hum Neurosci. 2013 Dec 10;7:836. doi: 10.3389/fnhum.2013.00836 (PMC3857923; doi:10.3389/fnhum.2013.00836)
Supplement: Supplementary file 1 [file Data_Sheet_1.PDF]

Supplementary Material

Questionnaire

E-mail: ____________________ Age: _____ years Sex: ( ) Male ( ) Female

1 – How often do you recall your dreams?

( ) Always = I recall my dreams every day

( ) Very frequently = I recall my dreams almost every day

( ) Frequently = I recall my dreams once or twice a week

( ) Rarely = I recall my dreams once or twice a month, approximately

( ) Very rarely = I recall my dreams once a year, or less

( ) I never recall my dreams (if you choose this answer, go to the end of the page and press "Send")

2 – At what time of the day you tend to dream?

( ) 06:00 - 12:00 ( ) 12:00 - 18:00 ( ) 18:00 - 00:00 ( ) 00:00 - 06:00 ( ) Don't Know

3 – What do you most remember about your dreams? "I dream with...". Check below according to the approximate frequency.

	Always	Very Frequently	Frequently	Rare	Very Rare	Never
Colored images						
Black and white images						
Sounds / Voices						
Smells						
Tastes / Flavors						
Known people						
Unknown people						
Something pleasant						

	Always	Very Frequently	Frequently	Rare	Very Rare	Never
Movements / Actions						
Natural Scenes						
Flying						
Sexual Intercourse						
Non-existent Creatures						
Someone who has died						
A mirror						
Things to read						
Other						

4 – Do you dream in first person (active dreams “from within”, in which you make decisions and act in the dream at will), or as third person (passive dreams in which you participate “from without” as an observer or spectator, "as in a movie," "watching yourself" as if you were just another character in the dream).

( ) Always in first person ( ) Usually in first person ( ) Always in third person ( ) Usually in third person ( ) Don’t know

5 – Some dreams appear mostly after an emotionally charged event in life, such as to dream with an accident after it really happens. Did this ever happen to you?

( ) Yes ( ) No

If you said Yes to the previous question, in which situation did it happen? You can check more than one answer.

[ ] After something bad happened (mugging, accident, natural disaster, sexual abuse, divorce, fights, discussions etc.)

[ ] After a friend/family member died I dreamed about him/her

[ ] After something good happened (job promotion, an award, meet a loved one, pass an examination etc.)

6 – Your dreams have to do mostly with:

( ) The previous day

( ) The last week

( ) The last month

( ) The last year

( ) Events more than one year older

( ) Plans for the next days

( ) Nothing related to me

7 – What is the content of your nightmares? Check below according to the approximate frequency.

	Always	Very Frequently	Frequently	Rare	Very Rare	Never
Anxiety/Fear						
Pain						
Other unpleasant sensations						
Being chased						
Chasing something						
Wars						
Monsters						
Ghosts / Spirits / Souls						
Threat to psychological integrity						
Threat to physical integrity						
Environmental disasters						
Frustration / Failure on a goal						
Other						

8 – Have you ever experienced repetitive dreams, i. e. dreams that appear with an equal (or similar) content in a recurrent way? Check below according to the approximate frequency.

	Always	Very Frequently	Frequently	Rare	Very Rare	Never
Falling tooth						
Being naked in front of other people						
Being late for an appointment						
Sex						
To dream that you are falling						
Other						

9 - Could you ever determine at will the content of your dreams? Check below according to the approximate frequency.

	Always	Very Frequently	Frequently	Rare	Very Rare	Never
If I think about something before sleeping I can dream about it						
When I wake up from a good dream I can return to it						
When I am aware of the dream during the dream						

10 – When you are dreaming, has some feature of the room / sleep environment ever entered the dream (stimulus incubation)? Check below according to the approximate frequency.

	Always	Very Frequently	Frequently	Rare	Very Rare	Never
Phone ringtone						
Alarm signal						
TV/Radio sounds						
House/Street noises						
Human voice						
A light						
A smell						
A tactile sensation						
Other						

11 - As bizarre as dreams are, usually we believe that what is happening in the dream is true. However, during a special kind of dream called lucid dream, one is sure to be dreaming during the dream, and may (or not) control the dream content. In other words, the dreamer can do whatever it wants in the dream. Please answer now some additional questions about lucid dreams. Did you ever hear about lucid dreams?

( ) Yes ( ) No

12 – Have you experienced lucid dream(s)?

( ) Yes ( ) No

If you marked Yes, answer the following questions. If you marked No, please go to the end of the page and press "Send".

13 – How old were you when you experienced your first lucid dream(s)?

( ) Under 5 ( ) 5 - 10 ( ) 10 - 15 ( ) 15 - 25 ( ) 25 - 50 ( ) After 50 years

14 – At which age you experienced more lucid dream(s)? You can check more than one answer.

[ ] Under 5 [ ] 5 - 10 [ ] 10 - 15 [ ] 15 - 25 [ ] 25 - 50 [ ] After 50 years

15 – How many lucid dream(s), approximately, have you ever experienced?

( ) Between 1-5
( ) Between 5-10
( ) Between 10-50
( ) Between 50-100
( ) More than 100
( ) Once a week
( ) Every (or almost every) day

16 - How long, approximately, are your LD episodes?

( ) They are very fast, I wake up as soon as I know I am dreaming
( ) Less than 10s
( ) Between 10s-1min
( ) Between 1-10min
( ) More than 10min
( ) The time I want

17 – Are your lucid dream(s) pleasant? Would you like to experience more LD?

( ) Yes, they are pleasant, and I would like to experience LD more often.

( ) Yes, they are pleasant, but I would not like to experience LD more often.

( ) No, they are unpleasant, and I would not like to experience LD more often.

18 - How often do you control your LD episodes (partial or full control)?

( ) Always ( ) Very Frequently ( ) Frequently ( ) Rare ( ) Very Rare ( ) Never

Whenever you are able control the LD, what do you usually do?

( ) Fly

( ) Have sex

( ) Meet deceased people

( ) Visit different places

( ) Other love situations

( ) Meet friends, family

( ) Have radical situations, sports etc

19 – At what time of the day you tend to have more lucid dream(s)?

( ) During naps ( ) During sleep ( ) After waking up and returning to the same dream

20 - When you have a lucid dream, which of the factors below usually influence the dream content? You can check more than one answer.

[ ] Think about / wish to have a lucid dream before sleep

[ ] Think about my dreams when I am awake (during the day)

[ ] Stress

[ ] Insomnia/Sleep deprivation

[ ] Be drowsy when awake

[ ] Too much work

[ ] Too much study

[ ] Physical activity/sports

[ ] Meditate

[ ] Sleeping without a fixed time to wake up

[ ] A food

[ ] A drug

[ ] A medicine

[ ] To travel

[ ] Have sex

[ ] A light sleep

[ ] A deep sleep
